# Leukocyte infiltration across the blood-spinal cord barrier is modulated by sleep fragmentation in mice with experimental autoimmune encephalomyelitis

**DOI:** 10.1186/2045-8118-11-27

**Published:** 2014-12-28

**Authors:** Junyun He, Hung Hsuchou, Yi He, Abba J Kastin, Pramod K Mishra, Jidong Fang, Weihong Pan

**Affiliations:** Blood-Brain Barrier Group, Pennington Biomedical Research Center, Baton Rouge, LA 70808 USA; Department of Biology, University of Texas, San Antonio, TX 78249 USA; Department of Psychiatry, Pennsylvania State University, Hershey, PA 17033 USA; BioPotentials Sleep Center, 8032 Summa Ave, Ste A, Baton Rouge, LA 70809 USA

**Keywords:** EAE, Leukocytes, Circadian rhythm, Sleep fragmentation, Spinal cord

## Abstract

**Background:**

We have recently shown that mice with experimental autoimmune encephalomyelitis (EAE) have increased sleep fragmentation (SF) and reduced sleep efficiency, and that the extent of SF correlates with the severity of disease. It is not yet clear whether and how sleep promotes recovery from autoimmune attacks. We hypothesized that SF promotes leukocyte infiltration across the blood-spinal cord barrier, impairs immune regulation, and thus worsens EAE.

**Methods:**

Three groups of C57 mice were studied: Resting EAE; SF EAE with the mice subjected to the SF maneuver 12 h /day during zeitgeber time (ZT) 0–12 h; and naïve controls with neither EAE nor SF. Besides monitoring of the incidence and severity of EAE, the immune profiles of leukocytes in the spinal cord as well as those in the spleen were determined.

**Results:**

When analyzed 16 days after EAE induction, at which time the SF was terminated, the SF group had a greater number of CD4^+^ T cells and a higher percent of CD4^+^ cells among all leukocytes in the spinal cord than the resting EAE group. When allowed to recover to 28 days after EAE induction, the SF mice had lower EAE scores than the resting EAE group. EAE induced splenomegaly and an increase of Gr1^+^CD11b^+^ myeloid-derived suppressor cells in the splenocytes. However, SF treatment had no additional effect on either peripheral splenocytes or granulocytes that reached the spinal cord.

**Conclusion:**

The SF maneuver facilitated the migration of encephalopathic lymphocytes into the spinal cord. Paradoxically, these mice had a better EAE score after cessation of SF compared with mice without SF.

## Background

Sleep is a restorative process for the biological system [1–3]. Disruption of sleep is generally thought to have adverse effects on the progression of autoimmune diseases, as seen in systemic lupus erythematosus [4] and rheumatoid arthritis [5]. In human subjects with multiple sclerosis (MS), treatment of sleep disorders improves symptoms of fatigue [6]. Experimental autoimmune encephalomyelitis (EAE) is an autoimmune disease targeting myelin components in the central nervous system (CNS). It serves as a useful autoimmune model for MS. While MS patients have many presentations of sleep disturbance, mice with EAE show mainly sleep fragmentation (SF) and reduced sleep efficiency. This is seen throughout the course of the symptomatic phase in the Friend Virus B-Type (FVB) strain of female mice, with the extent of sleep disruption correlating with EAE symptoms [7]. This has led to the hypothesis that sleep disruption worsens EAE. We tested this by a chronic experimental SF maneuver applied during the light span when mice typically would be asleep. This SF protocol has been shown to successfully increase sleep state transition and reduce non-rapid eye movement (NREM) sleep across the days of manipulation, with fast recovery after cessation of SF [8].

EAE is predominantly an autoimmune disorder involving hyperactive helper T cell (Th)-1 and Th-17 responses with CD4^+^ cells targeting the myelin components in the spinal cord and brain. Th1 cytokines, including interferon (IFN)-γ and tumor necrosis factor (TNF)-α, are also elevated in EAE [9]. Even in mice without autoimmune disease, there are dynamic interactions between leukocytes and cytokines and the blood–brain and blood-spinal cord barriers (BBB). For example, fibroblast growth factor-19 crosses the BBB by a non-saturable transport system [10], whereas TNF and leukemia inhibitory factor show regulatory changes in their saturable transport across the BBB [11–14]. Most recently, we showed that chronic sleep restriction induces multifaceted changes in BBB function, including an increase of paracellular permeability to small tracers, reduction of glucose uptake, and inflammation of the cerebral endothelia [15].

Thus, we postulated that sleep manipulation changes T cell activity and modifies the course of EAE. In healthy men, Th1 activity is highest in early nocturnal sleep, with increased production of IFN-γ and interleukin (IL)-4, whereas the Th2 response occurs during late sleep following release of prolactin and growth hormone. The production of TNFα by CD8+ T cells is decreased after sleep. The results indicate that there are distinct circadian rhythms of T cell activity with regard to Th1 and Th2 balance [16]. Therefore, we analyzed both CD4 and CD8 T cell populations in the spleen and spinal cord to determine the immunopathology of EAE mice in response to SF. In mice with an astrocytic leptin-receptor mutation and worsened EAE, we have shown a reduction in the percent of *Gr1*^*+*^*CD11b*^*+*^ subtype of granulocytes infiltrating the spinal cord [17]. Therefore, this population of leukocytes was also analyzed to test the hypothesis that this leukocyte population played a disease-modulating role in mice after SF.

## Materials and methods

### Animals and EAE induction

C57BL/6 J mice were obtained from Jackson Laboratory (Bar Harbor, ME, USA) and housed in the animal care facility for 1–2 weeks before induction of EAE at age 8–10 weeks, following a protocol approved by the PBRC Institutional Animal Care and Use Committee. Females were used because they are more susceptible to EAE. All mice were housed in groups of four in round acrylic sleep recording cages (Pinnacle Technology, Lawrence, KS, USA) and adapted for 3 days before initiation of SF or control experiments.

EAE induction was similar to that reported recently [7, 17, 18], with minor modifications. In brief, 80 or 100 μg of myelin oligodendrocyte glycoprotein (MOG) fragment 35–55 (MOG_35–55_) was fully emulsified in 100 μl of complete Freund’s adjuvant (CFA) containing 500 μg of heat-killed *Mycobacterium tuberculosis* H37RA (DIFCO Laboratories, Detroit, MI, USA). The mixture was delivered subcutaneously, divided among three flank areas. Pertussis toxin (Sigma, St. Louis, MO, USA) was injected intraperitoneally immediately after induction and then again 48 h later. The time of induction was considered EAE day 0. Symptoms were monitored daily at about noon (zeitgeber time, ZT6) by use of a standard EAE scoring system [11, 19, 20], with 0 being symptom-free and 5 being the worst (moribund or dead).

### Experimental SF maneuver

Three groups of mice were used (n = 8 /group): (1) EAE mice with SF during the light phase from day −10 to day +16 in relation to EAE induction (on day 0). SF was applied between zeitgeber time (ZT) 0–12; (2) resting EAE mice without SF intervention; (3) naïve controls with neither EAE nor SF. The dose of MOG_35–55_ was 100 μg/mouse. The mice were sacrificed on day 16 after EAE induction, immediately after cessation of SF.

In a second study to determine the progression of EAE symptoms, 2 groups of mice were studied (n = 8/group): SF EAE mice and resting EAE mice, as for first group above. In this case, EAE scores and body weight were monitored from day 0 till day 28 after EAE induction. The dose of MOG_35–55_ was reduced to 80 μg/mouse: by reducing the dose of the heptagen, we aimed to achieve a lower EAE score in this batch of mice. This would ensure that mice were not too incapacitated to have the SF procedure and that they could be monitored through the course of EAE to 28 days. This would increase the probability of identifying the effect of SF, as we hypothesized that SF worsens EAE.

The SF maneuver agitated the mice every 2 min by use of random bar rotation driven by a computer program. The mice were group-housed in round cages (3 – 4 mice/cage). At the bottom of the cage is a metal bar slightly shorter than the inner diameter of the round cage and positioned above the corncob bedding. A computer-controlled SF schedule repeats on a 120 s cycle (30 s on, 90 s off) during the light span (6 am to 6 pm, ZT0-12), with an intermediate bar rotation speed (scale of 5 out of 10). The direction of bar rotation is randomly reversed every 10–40 sec. The choice of 30 events/h of SF used was based on clinical evidence from severe sleep apnea, and shown to be effective in compromising sleep architecture in mouse and rat [21, 22]. The effective reduction of NREM sleep and increased sleep fragmentation with this SF maneuver has been validated by sleep recording and sleep architectural analysis, as we have reported recently [8]. In brief, SF increases Wake time by about 25% in the 12-h light span, reduces % NREM by about 20%, but decreases REM sleep only during the first night. The changes are consistent across the 10 d of SF despite the reduction of REM sleep for the first night. By contrast, control C57 mice have about 39% Wake, 56% NREM, and 5% REM. The SF maneuver also increases sleep state transitions: NREM bouts increase 2–3 fold, as do Wake bouts. In the dark span, the percent of Wake, NREM, and REM show no significant changes, and there is no increase of sleep fragmentation. This indicates a lack of circadian shift resulting from the SF maneuver. Recovery from SF is rapid, as sleep architecture returns to baseline within 24 h after the cessation of SF [8].

### Flow cytometry

Three groups of mice were studied (n = 8 /group): naïve, resting EAE, and SF EAE where SF was applied 10 days before EAE induction and terminated on day 16 of EAE, immediately before sacrifice of the mice in the afternoon (ZT6-9). To isolate leukocytes from the spinal cord and spleen following an established protocol [23], mice were anesthetized by intraperitoneal injection of urethane (30 mg/kg). They were perfused intracardially with phosphate-buffered saline (PBS) to remove leukocytes in the spinal cord vasculature. The spinal cord was homogenized in PBS containing 0.1% fetal bovine serum. Leukocytes were recovered at the 30:70% Percoll interface after gradient centrifugation as described previously [17]. To obtain splenocytes, spleens were ruptured, homogenized, filtered through a 40 μm nylon mesh, centrifuged, and subjected to red blood cell lysis. Cells from both tissues were washed with FACS buffer twice, immunostained, fixed with 2% paraformaldehyde (PFA), and stored in PBS until flow cytometric analysis.

All antibodies were purchased from BioLegend (San Diego, CA, USA). For cell surface staining of immune markers, leukocytes were blocked with anti-mouse CD16/32 antibody (Clone 93, catalog number 101302), incubated at 4°C with FITC-conjugated CD4 (Clone GK1.5, catalog number 100405), PE-conjugated CD8a (Clone 53–6.7, catalog number 100707), Alexa488-conjugated CD11b (Clone M1/70, catalog number 101219), APC-conjugated Gr1 (Clone RB6-8C5, catalog number 108411), or APC-conjugated CD45 antibody (Clone 30-F11, catalog number 103112). Control conditions included single staining for antibodies and non-stained cells to exclude autofluorescence. After staining, the cells were fixed with 2% PFA and stored in PBS for 1–3 days. Cell numbers were estimated by use of a hemocytometer, and the immunofluorescent intensity was analyzed by FACSCalibur (BD Pharmingen, San Diego, CA, USA). Data were analyzed with post-collection compensation by FlowJo (Tree Star, Ashland, OR, USA) software.

The gating strategy was as follows: Cells were first gated based on side scatter of CD45 immune positive cells, which differentiates plasma cells, non-lysed erythrocytes, and their precursors from the CD45^+^ leukocytes. Blast cells and other hematogenous cells with low granularity and low CD45 intensity were located in the left lower quadrant of the side scatter plot of the CD45^+^ population, whereas lymphocytes, which show high CD45 intensity and low granularity, were gated in the right lower quadrant. Granulocytes, including neutrophils of relatively lower CD45 and eosinophils with higher CD45 immunofluorescence, were gated in the upper right quadrant of the side scatter plot. Monocytes with high CD45 and intermediate granularity were separated from the small number of basophils that had lower granularity. The number of lymphocytes was then used as the denominator to determine the percentage of CD4 and CD8 T cells in double immunostaining. The percentage of CD4 and CD8 cells among all leukocytes was also determined. Although some macrophages also express CD4 and CD45 and some dendritic cells also express CD8 and CD45 besides lymphocytes, these two populations were excluded with gating for the final analysis. For Gr1 and CD11b double labeling, there were four clearly defined quadrants representing Gr1^+^CD11b^+^ myeloid-derived suppressor cells, Gr1^−^CD11b^+^ macrophages, monocytes and dendritic cells.

### Statistical analysis

Means were expressed with their standard errors. Repeated measures analysis of variance (ANOVA) was used to determine the effect of EAE and SF on EAE scores. One-way ANOVA was used to determine the effect of SF and EAE on spleen weight and leukocyte populations, followed by Tukey’s post-hoc test. Student’s *t*-test (2-tailed) was used when only two groups were present for comparison. Prism GraphPad 5 statistical and graphic program (GraphPad, San Diego, CA, USA) was used for statistics and graphics.

## Results

### Effect of SF on EAE scores

**Figure 1:**
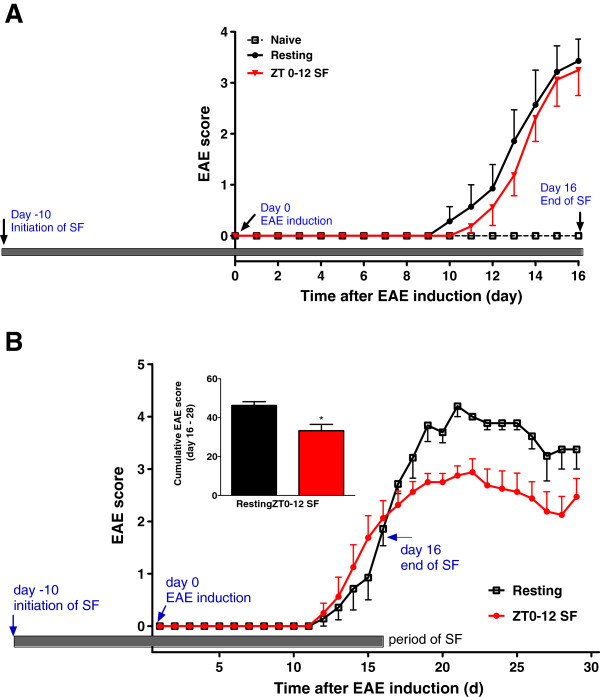
**Effect of sleep fragmentation (SF) on EAE scores and disease progression.** SF was applied from 10 days before EAE induction until 16 days after induction. **(A)** Three groups of mice (n = 8 /group) were studied: without induction of EAE (naïve), induction of EAE with 100 μg /mouse of MOG_35–55_ without SF (resting) and with SF (ZT 0–12 SF). By the end of the study on day 16, there was no difference in EAE score between the resting EAE group and the SF EAE group (ZT0-12 SF). **(B)** Two groups of mice (n = 8/group) with EAE induction with 80 μg/mouse of MOG_35–55_ were monitored until day 28. In the SF EAE group (ZT 0–12 SF), the EAE scores were lower in the recovery phase (Day 16–28) than resting EAE, shown both by the daily progression and the cumulative score (inset). *: *p* < 0.05.

The incidence rate of EAE was 100% in all EAE groups at both 100 and 80 μg/mouse doses of MOG_35–55_. For both doses, the SF maneuver was initiated 10 days before EAE induction and terminated 16 days after EAE induction. In the first experiment with 100 μg of MOG_35–55_, the resting EAE mice seemed to show a slightly earlier onset of symptoms at day 10 compared with the SF mice on the same day. However, this was not statistically significant and there was no difference between the EAE groups at day 16 (Figure 1A). Since we hypothesized that SF will worsen EAE, SF was expected to increase the EAE score from the control (non-SF) group to a higher score (greater severity). However, we considered that any effect of SF might have been masked by the severe EAE in mice receiving the higher dose (a score of about 3.5 in comparison with a score of 2 at the lower dose on day 16). In this case, using mice that achieved a lower basal score would be more likely to identify a potential effect.

To further test the hypothesis that SF worsens EAE, the second study used a lower dose of MOG_35–55_ (80 μg/mouse). In the resting EAE group, EAE symptoms emerged on day 12, gradually increased in severity, and reached a peak on day 21. These mice were allowed to recover until day 28 at which time they were euthanized along with resting EAE and naïve mice. At the lower dose of MOG_35–55_ (80 μg/mouse), there was a significant effect on EAE score of SF [F (1,348) = 40.6, *p* < 0.001] and of days after EAE [F (28,348) = 72.1, *p* < 0.001], and their interaction was also significant (*p* < 0.001). The average score from Day 16 to Day 28, derived from analysis of the area under curve, was significantly reduced in the mice with previous SF (*p* < 0.05) (Figure 1B, inset). Overall, the cessation of SF with 12 days of recovery stabilized the EAE score with improvement over the resting (non SF) EAE group (Figure 1B).

### SF increased leukocyte infiltration into the spinal cord compared with the splenocyte immune profile

**Figure 2:**
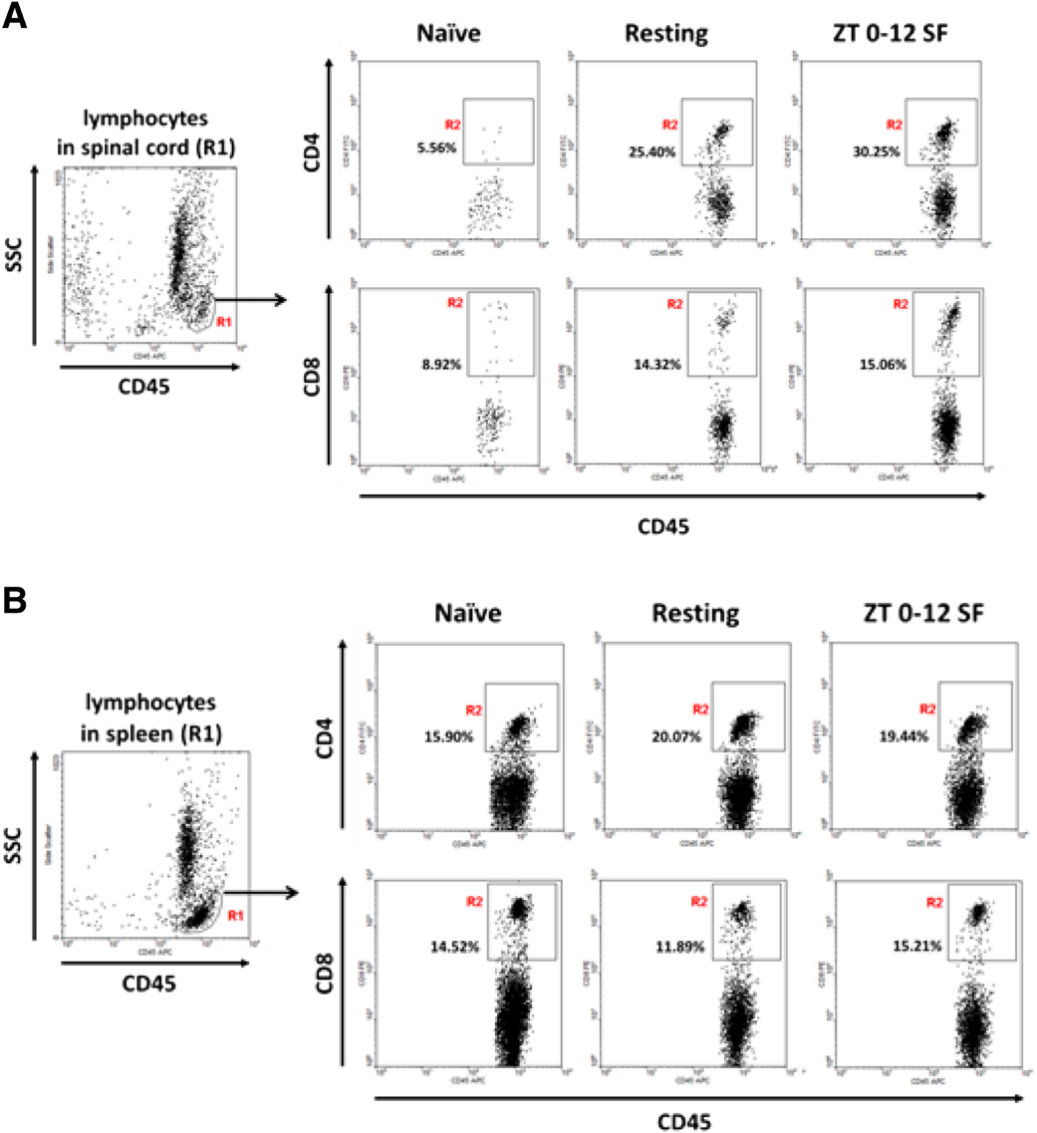
**Displays showing the PanLeucogating method and group differences of CD4 and CD8 cells in cells recovered at day 16 from homogenized spinal cord (A) and spleen (B) in naïve, resting EAE and SF EAE (ZT 0–12 SF) mice (dose = 100 μg/mouse).** SSC: side scatter.

**Figure 3:**
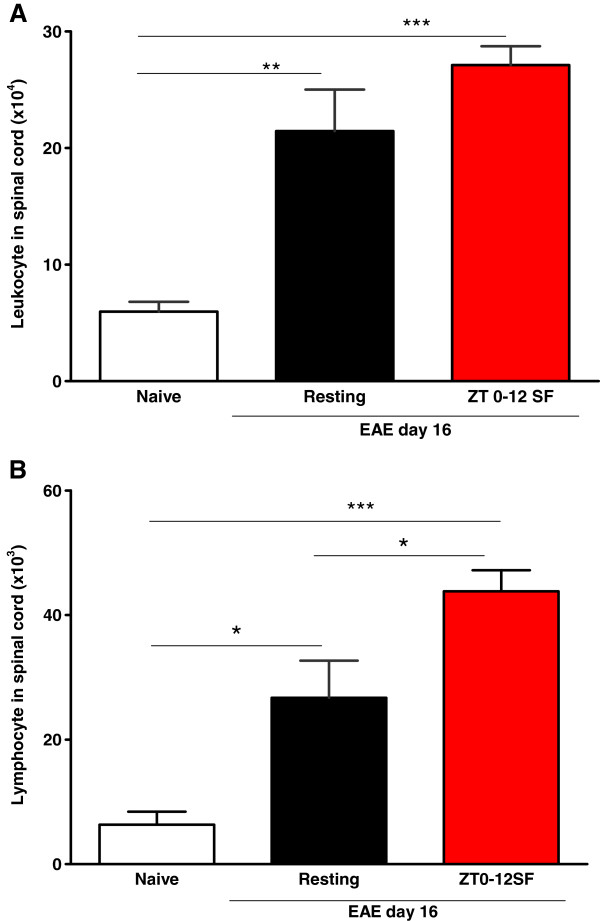
**Histograms showing both resting EAE and SF EAE (ZT 0–12 SF) mice on day 16 showed an increased infiltration of (A) leukocytes (CD45** ^**+**^ **cells) and (B) the lymphocyte population (low granularity and high CD45 immunofluorescent intensity) in the spinal cord (n = 8 /group).** *: *p* < 0.05; **: *p* < 0.01; ***: *p* < 0.005.

To test the hypothesis that SF is associated with an increase in the infiltration of leukocytes, particularly lymphocytes, across the blood-spinal cord barrier, leukocytes from the spinal cord and splenocytes from the spleen were subjected to flow cytometry after extraction on day 16 after induction in the three groups of mice: naïve, resting EAE, and SF EAE (100 μg/mouse). Populations of leukocytes from spinal cord (Figure 2A) and spleen (Figure 2B) were gated based on CD45 positivity on side scatter plots as described in Methods above, and further differentiated into CD4 and CD8 (+) populations after dual immunostaining. One-way ANOVA showed that EAE increased the infiltration of CD45^+^ leukocytes in both EAE groups [F (2, 15) = 20.05, *p* < 0.001] (Figure 3A) although the results did not show a significant difference when the SF group was compared with the resting EAE mice. This negative finding was important and contrasts with an increase of the infiltrating CD4 T cells shown below. For the lymphocyte subpopulation, EAE resulted in a greater increase in infiltration compared with the naïve mice [F (2, 16) = 15.9, *p* < 0.001] (Figure 3B). SF EAE had more infiltrated lymphocytes than resting EAE (*p* < 0.05).

**Figure 4:**
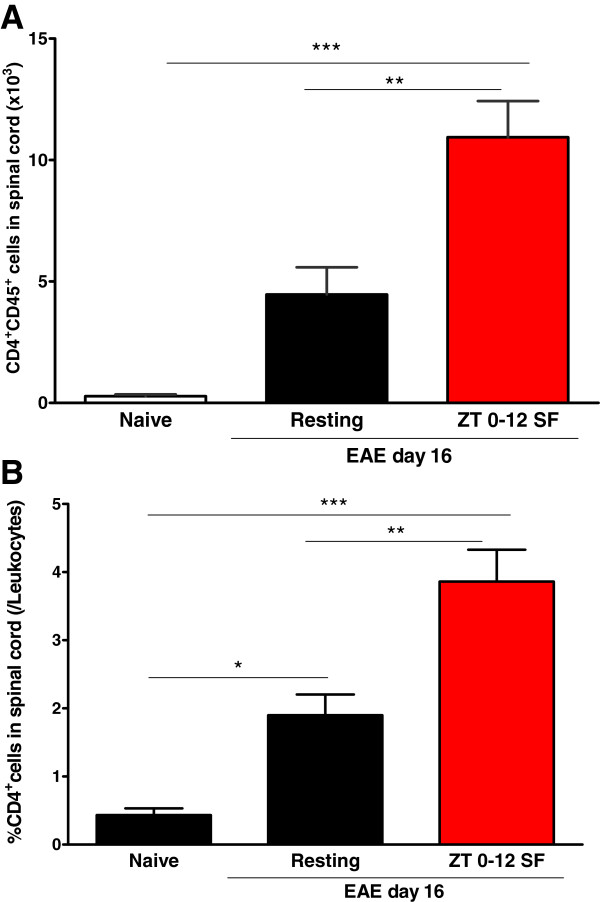
**Histograms showing that SF EAE (ZT 0–12 SF) induced an increase over resting EAE mice in (A) the total number of CD4** ^**+**^ **T cells and (B) the percent of CD4** ^**+**^ **T cells among all leukocytes infiltrating the spinal cord on Day 16 of EAE (n = 8 /group).** *: *p* < 0.05; **: *p* < 0.01; ***: *p* < 0.005.

The total number of CD4^+^ T cells in the spinal cord was higher in the SF EAE mice than the resting EAE group [F (2, 14) = 20.46, *p* < 0.001) (Figure 4A). The percent of CD4^+^ cells among all infiltrated leukocytes was also increased [F (2, 15) = 22.02, *p* < 0.001] (Figure 4B). This contrasts with a lack of increase in the percent of CD8+ cells or Gr1^+^CD11b^+^ cells in the post-hoc Tukey’s test, despite a significant overall effect of EAE shown by ANOVA (data not shown).

### SF did not increase Gr1^+^CD11b^+^ cells among splenocytes

**Figure 5:**
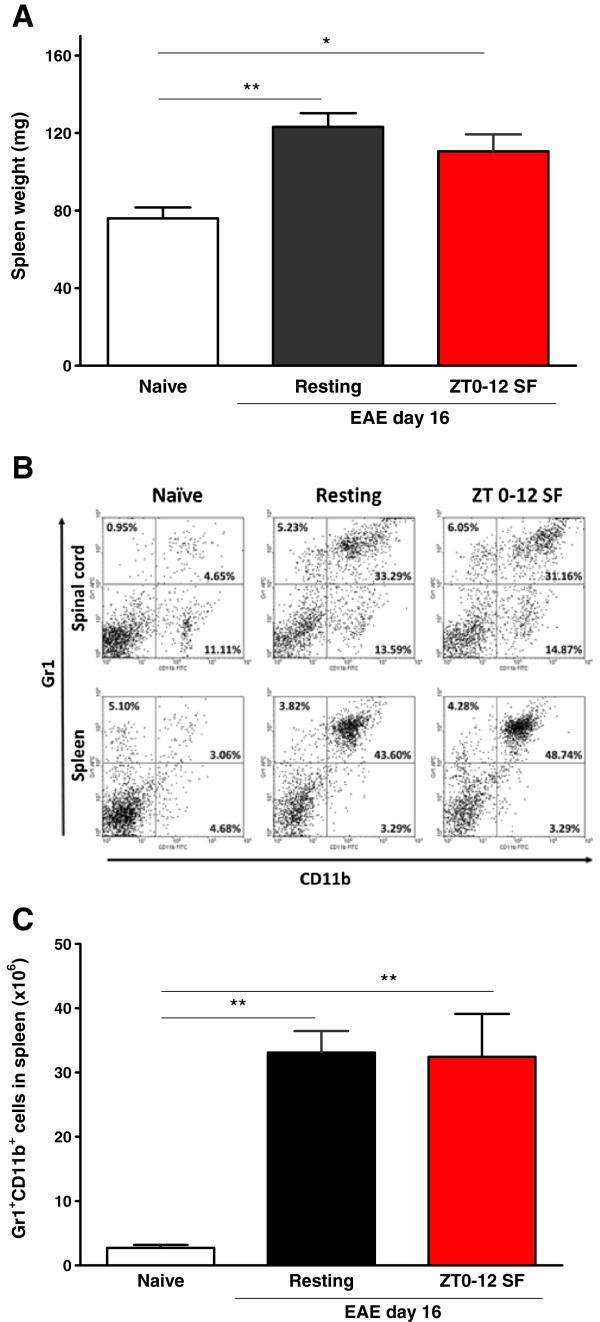
**Effects of EAE on the spleen in mice at day 16 after induction: SF had no additional effect besides that of EAE to modulate peripheral immune cell proliferation (n = 8 /group). (A)** EAE induced significant splenomegaly. **(B)** Gating of Gr1 and CD11b (+) populations with double labeling. **(C)** The total number of Gr1^+^CD11b^+^ cells was increased by EAE but SF had no additional effect. *: *p* < 0.05; **: *p* < 0.01.

That EAE activates the immune system was shown by increased splenic weight in EAE mice [F (2, 15) = 9.01, *p* < 0.01] despite all showing significant body weight loss (Figure 5A). Coinciding with the reduction of the number as well as the percent of CD4^+^ and CD8^+^ T cells, the Gr1^+^CD11b^+^ myeloid-derived suppressor cells were increased in the spleen of EAE mice [F(2, 16) = 11.34, *p* < 0.001]. The gating of the cells and representative flow cytometric patterns are shown in figure 5B. SF had no additional effects on the Gr1^+^CD11b^+^ splenocytes besides EAE (Figure 5C).

## Discussion

Leukocyte infiltration into the CNS involves complex interactions between leukocyte antigens and the barriers of the CNS. Immune cells cross the BBB by a regulated process termed diapedesis. Results from this study showed that SF increased the percentage of leukocytes, particularly lymphocytes, infiltrating the spinal cord on day 16 of EAE, a time when the immune reaction and interactions among infiltrated cells and CNS residential cells were both active. In comparison with the resting EAE group, SF was associated with more CD4^+^ T cell infiltration into the spinal cord. There was also a higher percent of CD4^+^ T cells among all infiltrated leukocytes. This contrasts with a lack of additional effect of SF on EAE-induced splenomegaly and increased granulocytes in the spleen or spinal cord. Overall, the results suggest that an exaggerated CNS autoimmune response is typically associated with worsening of EAE [17, 18].

Sleep-wake activity also modulates regulatory T (Treg) cells in healthy men; natural Treg cell counts and their suppressive activity appear to be highest during the night and lowest during the day. Sleep deprivation not only abrogates the rhythm of CD4^+^CD25^+^ Tregs but also reduces the proliferation of CD4^+^CD25^−^ naïve T cells [24]. In EAE mice most of the NREM sleep loss and sleep fragmentation occurs in the light span (when the mice typically would be asleep), and there are dynamic changes at different stages of EAE [7]. This provided a basis for the application of SF in the light phase. Although we did not detect a circadian shift of sleep-wake cycle in naïve SF mice [8], the presence of recovery sleep in the dark phase and a circadian shift of sleep-wake activity cannot be ruled out in the SF EAE mice. These factors may have contributed to the circadian patterns of encephalopathic T cells and cytokines, as well as their neuroendocrine regulation.

Even in the absence of EAE, sleep disturbance with increased fragmentation and loss of about 20% of daily sleep for 6 days is sufficient to affect BBB function. There is increased paracellular permeability to sodium fluorescein, and higher uptake of biotin in certain regions that might involve both increased transport and increased BBB permeability, reduction of glucose uptake across the BBB, decreased vascular reactivity as well as regulatory changes of a series of transport-related proteins, and increased BBB inflammation [15]. As the result of immune cell infiltration and proliferation, the CNS undergoes inflammation, reactive gliosis, demyelination, and neuronal damage, producing distinct phases of EAE. The course of progression and recovery can be collectively reflected by behavioral scores, immune profiling, BBB damage, and morphological and biochemical changes of the CNS. These aspects can be regulated by sleep and its disorders as a result of alterations of hormones and neurotransmitters, and their circadian rhythms.

## Conclusion

In summary, we showed that SF increased the percentage of lymphocytes infiltrating the spinal cord on day 16 of EAE, particularly the number and percent of CD4^+^ T cells among all leukocytes. Overall, the results suggest a modulatory effect of SF on EAE progression. A better understanding of the effects of SF on EAE and the underlying mechanisms is essential, as this may lead to new strategies to maximize sleep treatment for optimal neurological function in patients with MS.

## Abbreviations

SF: Sleep fragmentation
NREM: Non rapid eye movement
REM: Rapid eye movement
EAE: Experimental autoimmune encephalomyelitis
ZT: Zeitgeber time.
